# *RSC Chemical Biology* Emerging Investigators Collection and Outstanding Paper Award

**DOI:** 10.1039/d2cb90027k

**Published:** 2022-08-01

## Abstract

This collection showcases research carried out by internationally recognised, up-and-coming scientists in the early stage of their independent careers who are making outstanding contributions to their respective fields.
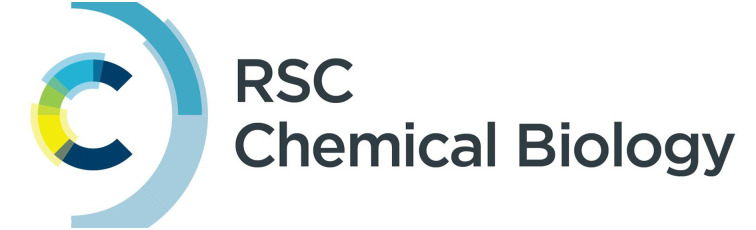

*RSC Chemical Biology* is committed to supporting and recognizing the excellent work of early career researchers. We are thus proud to present our first annual Emerging Investigators collection. The collection showcases research carried out by internationally recognised, up-and-coming scientists in the early stage of their independent careers who are making outstanding contributions to their respective fields. Each contributor was recommended by an expert in their field for carrying out work with the potential to influence future directions. The collection encompasses the entire scope of the journal.

We would like to invite you to read through the collection where you will find fascinating science ranging from drug development, enzyme structural studies, and improvements to peptide solid phase synthesis, to click chemistry for single-cell sequencing, labelling molecules in live cells and proteomic studies of macrophages. The variety of the themes covered in the Emerging Investigators collection represents the inclusiveness and broad range of the journal scope. Our emerging authors are clearly the breaking wave of creative new science and applications, and it is a privilege to be a part of their journey.

The papers accepted into the collection will be considered by the *RSC Chemical Biology* Editorial Board for one of the two Outstanding Paper Awards. In early 2023 the Editorial Board will choose one winner from among the papers accepted to the special collection in 2022. The winner will be announced in an editorial, invited to write a Perspective, and present their work in the webinar organized by *RSC Chemical Biology*.

Looking forward, we will soon be inviting for an Emerging Investigators Collection that will be published in 2023, and you are encouraged to recommend a colleague at the beginning of their career by contacting the journal’s Editorial Office at chembio-rsc@rsc.org. To be considered, a scientist must be a research group leader with less than 10 years of independent research (although the timescale is flexible in cases of career breaks and personal circumstances).

Congratulations to all the featured researchers on their work. Join us in celebrating their contributions!

## Supplementary Material

